# 

**Published:** 1882-04

**Authors:** 


SURG GENLS OFFS

Vol. XIX. April 1882. No. 1.

### The Bistoury.

Thad S. Up de Graff, M.D., Editor.

Elmira, N.Y.

